# Driving Implementation‐Focused Change for Gender Equity in Medical Leadership: A Consensus‐Based Priority‐Setting Approach

**DOI:** 10.5694/mja2.70314

**Published:** 2026-10-06

**Authors:** Belinda Garth, Jenny Proimos, Thuy Vy Nguyen, Sally Cross, Sarah Whitelaw, Helena J. Teede

**Affiliations:** ^1^ Monash Centre for Health Research and Implementation Monash University Melbourne Victoria Australia; ^2^ Australian Medical Association Canberra Australian Capital Territory Australia; ^3^ Royal Melbourne Hospital Melbourne Victoria Australia; ^4^ Monash Health Melbourne Victoria Australia

**Keywords:** medical colleges, qualitative research, surveys and questionnaires, women physicians

## Abstract

**Objectives:**

To identify evidence‐informed drivers of sustainable systems change and actionable organisational priorities for advancing gender equality in medical leadership, aligned with national and international policy mandates, through the perspectives of key medical interest‐holders.

**Study Type:**

Co‐production implementation approach using mixed‐methods, modified Delphi surveys and a Nominal Group Technique workshop. Reporting follows the REPRISE guideline for priority‐setting of health research.

**Setting, Participants:**

From September 2021 to August 2023; online interviews, member survey, pre‐workshop survey and a hybrid workshop with the Australian Medical Association (AMA). Purposive sample of AMA members, including doctors in training, doctors and medical leaders across all Australian states and territories.

**Main Outcome Measures:**

Consensus‐based priorities to inform a co‐produced implementation plan for gender equality in medical leadership.

**Results:**

Seventeen medical leaders were interviewed and 542 members completed the survey. Both consistently prioritised: (i) women's representation in leadership; (ii) inclusive and transparent recruitment; (iii) mentoring and networking; (iv) flexibility and family‐friendly policies; and (v) gender targets and quotas. Triangulated findings identified eight internal and three external opportunity areas. A priority‐setting workshop with 22 leaders generated 10 consensus priorities: five internal organisational (gender equity education; transparent leadership selection including gender targets; monitoring and reporting; promoting respect and monitoring behaviour at meetings/events; and leadership development for emerging women leaders) and five external (committed leadership for gender equity in medicine; advocacy for data and reporting; flexible career pathways; recognition of mentoring; and strategies to address the gender pay gap).

**Conclusion:**

Through a collaborative, evidence‐informed process, we coproduced consensus‐based priorities to advance gender equality in medical leadership with a leading national professional association that influences health policy. This structured approach is adaptable to other organisational contexts seeking to implement equity‐focused interventions. The resulting priorities have directly informed an action plan and implementation strategy underway to promote gender equity within the association and across the broader medical profession.

## Plain Language Summary

**The known:** Gender inequity persists in healthcare leadership and policy mandates call for systemic, evidence‐informed interventions.

**The new:** Through a mixed‐methods, consensus‐driven process guided by implementation science and interest‐holder engagement, medical association members identified 10 actionable priorities: five for implementation within the professional association and five focused on advocacy and support across the broader medical profession.

**The implications:** These priorities offer a practical and scalable roadmap for advancing gender equity in healthcare leadership. They informed adaptable implementation strategies now being implemented and evaluated, supporting systemic change aligned with national and international equity mandates.

## 1 Introduction

Women comprise about 70% of the global healthcare workforce and 75% in Australia, yet remain under‐represented in leadership and decision‐making roles [1, 2, 3]. Despite being one of the largest and most feminised sectors, healthcare continues to offer slow and unequal career progression to leadership for women. Gender equality in leadership is an important health‐system priority associated with workforce health, resilience and organisational effectiveness [4]. Evidence shows that diversity in leadership representation yields broad organisational and societal benefits, with women leaders positively influencing financial performance, innovation, engagement with ethical initiatives, maternal and public health outcomes, workplace culture and the advancement of other women [1, 5, 6, 7, 8]. Persistent inequality in leadership highlights the need for systemic and sustained action.

In this context, we define gender as a social and cultural construct encompassing differences in identity, expression and lived experience among women, men and non‐binary people [9]. We define women as all those who identify as women, consistent with current evidence [10]. Gender equity refers to the processes and practices required to achieve gender equality, which is the ultimate outcome [11, 12]. Advancing gender equity in healthcare leadership aligns with national legislation [13, 14, 15], global mandates such as the United Nations Sustainable Development Goals [16] and national initiatives such as the Working for Women Strategy [17].

While women increasingly step into senior roles, systemic barriers persist [1, 2, 18]. System‐ and organisation‐level interventions are important to dismantle these barriers and create inclusive leadership pathways [19, 20]. With slow progress and resource constraints, health service leaders and professional bodies may benefit from prioritising evidence‐informed, feasible actions to drive meaningful change.

Organisation‐level implementation of gender equity initiatives in healthcare leadership has often been ad hoc, inconsistent and insufficiently structured. Many initiatives are neither grounded in evidence nor evaluated for impact, limiting their effectiveness and sustainability [21, 22, 23]. Formal, evidence‐informed priority‐setting processes offer a structured means of building engagement, identifying feasible and high‐impact interventions and supporting organisational leaders and members in consensus decision‐making [24], yet are rarely applied with explicit implementation considerations in this context [24, 25, 26, 27].

As part of the National Health and Medical Research Council (NHMRC) and partner co‐funded Advancing Women in Healthcare Leadership (AWHL) initiative [28], we are collaborating with the Australian Medical Association (AMA), a lead partner, to co‐produce, implement and evaluate evidence‐informed system and organisational strategies to advance gender equity in medical leadership. This article reports the initial phase of this initiative, focused on priority identification.

Specifically, we aimed to:

- examine contextual factors, organisational practices and evidence‐informed strategies to inform tailored systems‐ and organisational‐level gender equity initiatives within the AMA; and
- coproduce a set of contextually relevant, evidence‐informed priorities for advancing gender equity in medical leadership, based on importance and feasibility and designed for practical implementation within short‐ and medium‐term timeframes.

By identifying system‐ and organisational‐level priorities, this study provides a foundation for subsequent implementation and evaluation and is intended to benefit doctors and medical leaders, as well as the broader medical profession and health system. The prioritised interventions are directed toward decision‐makers within the AMA (internal priorities)—including federal and state leadership, committees and governance bodies—and others with influence over medical leadership and workforce equity (external priorities).

## 2 Methods

### 2.1 Setting

This study was conducted as part of the AWHL National Partnership Initiative, led by the Partnership Centre for Gender Equality and Leadership Advancement, hosted by the Monash Centre for Health Research and Implementation (MCHRI) and Monash University. AWHL aims to co‐produce, implement, evaluate and scale system‐, organisational‐ and individual‐level interventions to advance gender equity in healthcare leadership.

The AMA is a national, voluntary member‐based professional organisation representing doctors and medical students in Australia. It advocates on behalf of its members on professional, ethical, workplace and public health issues and works closely with governments to inform and influence health policy. The AMA also undertakes policy development, advocacy and negotiation of industrial agreements on behalf of the medical profession and healthcare system. In 2021, the AMA represented 28,973 members, making it the largest voluntary association of doctors in Australia [29].

### 2.2 Study Design

We used a co‐production, mixed‐methods implementation approach, incorporating qualitative research and a structured, consensus‐based priority‐setting process. This work was guided by the Monash Systems Change Approach, integrating established implementation and evaluation frameworks, including the Consolidated Framework for Implementation Research (CFIR), the Learning Health System (LHS) and the Reach, Effectiveness, Adoption, Implementation and Maintenance (RE‐AIM) frameworks [30, 31, 32]. A multi‐step co‐production process integrated evidence from research, interest‐holders [33] and organisational practice (Aim 1), culminating in a modified Delphi process combined with a Nominal Group Technique (NGT) workshop [24] to reach consensus on feasible, evidence‐informed interventions (Aim 2; Figure 1). To ensure priorities reflected evidence gaps, we incorporated findings from systematic reviews and syntheses alongside input from the multidisciplinary research team and organisational leaders throughout the Delphi and workshop stages. Details of the broader program methods have been published elsewhere [28].

**FIGURE 1:**
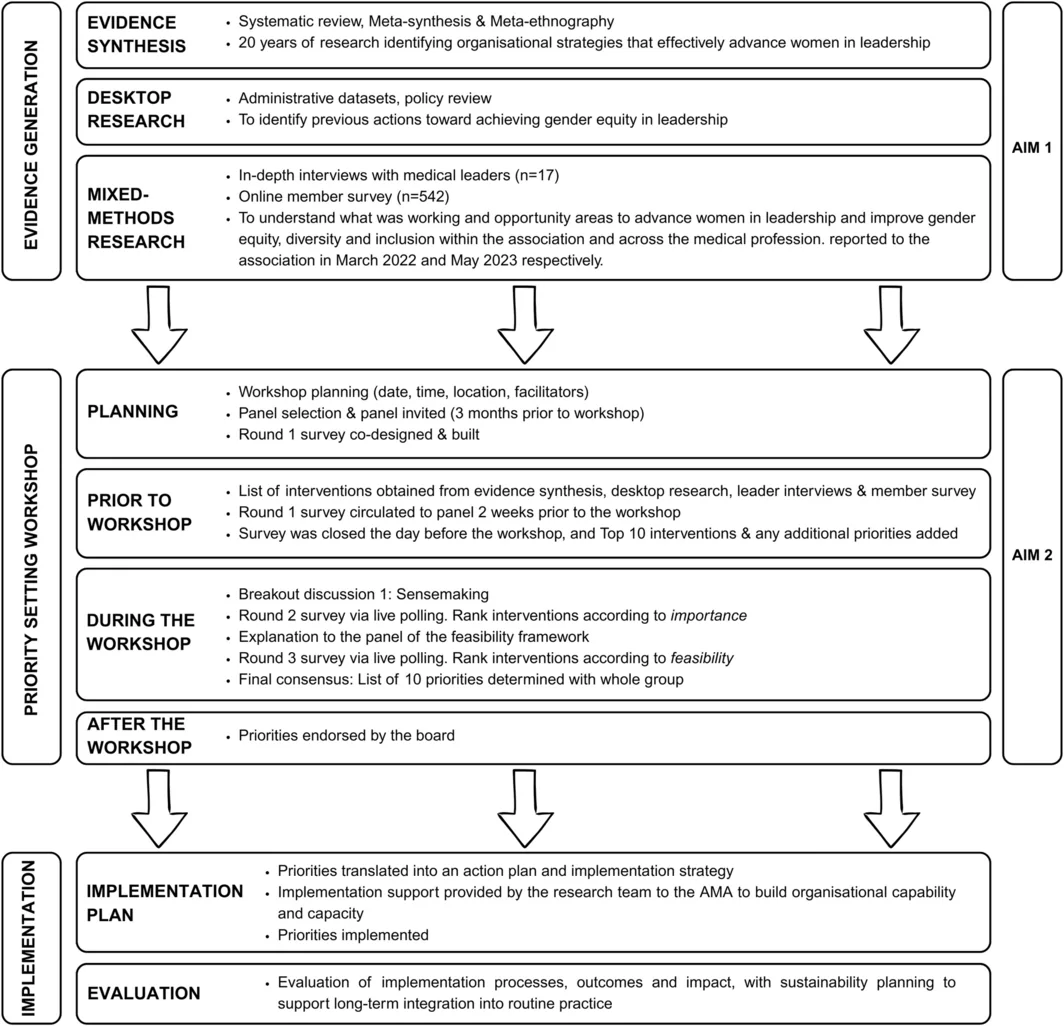
Co‐production approach integrating evidence synthesis, organisational review, interviews and survey findings to identify priorities that inform implementation planning and subsequent evaluation.

### 2.3 Mixed‐Methods Research (Aim 1)

#### 2.3.1 Data Collection

A purposive sample of 38 doctors and medical leaders who currently or previously held representative roles within AMA councils, committees or the presidency were invited to participate in semi‐structured interviews. Invitations were distributed by the AMA via an expression‐of‐interest process and interviews were conducted with all individuals who indicated willingness to participate. Interviews were conducted online between September and December 2021, audio‐recorded, transcribed verbatim and de‐identified.

In addition, an online survey of AMA members (including doctors and doctors in training) was conducted to assess organisational culture, perceived effectiveness of gender equity strategies and gaps in practice. The interview guide and survey were informed by published evidence [21, 23] and co‐produced by the research team and the AMA. The survey included predominantly closed questions with some open‐ended questions. Invitations to participate were via the AMA's newsletter and direct email, with additional promotion through social media. The survey was open from February to November 2022. Participation was voluntary and uncompensated.

#### 2.3.2 Data Analysis

Interview transcripts and qualitative survey responses were analysed using template analysis [34]. An initial coding template informed by the study aims and evidence synthesis [21] was organised around six domains: (i) leadership commitment and accountability; (ii) organisational practice, process and policy; (iii) organisational culture, awareness and engagement; (iv) organisational support tools; (v) mentoring and professional networks; and (vi) leadership training and development. The template was developed by a research assistant through coding an initial subset of transcripts and iteratively refined through team discussion until consensus was reached. Two sub‐codes were applied within each domain: factors supporting gender equity in leadership and opportunities for improvement. The framework was then applied across the dataset, with inductive coding used to identify additional concepts and refine the template where required. Dedoose 10.0.35 was used to manage and code the data. Quantitative survey data were analysed descriptively.

### 2.4 Priority‐Setting Process (Aim 2)

#### 2.4.1 Participant Recruitment

Individuals were eligible to participate in the priority‐setting process if they held leadership roles within the AMA. A purposive sampling approach was used to identify leaders across state and federal jurisdictions, career stages and genders. Fifty‐four leaders (26 women, 28 men) were invited via email to participate in a modified Delphi process and workshop. Participation was voluntary and uncompensated.

#### 2.4.2 Modified Delphi Survey (Round 1)

A modified Delphi survey was developed by the research team in collaboration with the AMA, drawing on findings from the evidence synthesis and qualitative research (Survey S1). The survey was distributed confidentially to invited participants about 2 weeks before the workshop (July 2023). Participants were asked to prioritise proposed interventions to advance gender equity in medical leadership across two domains: internal organisational action and external advocacy across the medical profession. Mean ranking scores were calculated for each item, and participants could suggest additional priorities. The top 10 ranked items in each domain were carried forward to the workshop, along with any additional suggestions. Items ranked outside the top quartile in Round 1 were excluded a priori, unless reintroduced during sense‐making discussions. Additional suggested priorities were included only if they met predefined scope (advancing gender equity in medical leadership) and feasibility criteria.

#### 2.4.3 Nominal Group Technique Workshop (Rounds 2 and 3)

A 4‐h hybrid workshop was held at the AMA federal office in Canberra, Australian Capital Territory, in August 2023 and facilitated by the research team. Round 1 survey results were presented to participants to inform structured discussion and sense‐making. Small‐group discussions focused on consolidating, clarifying or reintroducing priority areas, followed by confidential live ranking of interventions using the Poll Everywhere tool (https://www.polleverywhere.com/) based on perceived importance (Round 2). Separate polls were conducted for internal and external priorities.

A feasibility framework (Table S1) was used to guide discussion based on practical implementation considerations and a final ranking of proposed priorities followed (Round 3). Interventions ranked outside the predefined threshold of 10 items in Round 1 were excluded unless reintroduced through group discussion. After Round 3 voting, the ranked priorities were reviewed in a final facilitated discussion, allowing participants to clarify and confirm the final consensus‐based priorities for implementation.

Following the workshop, a summary report of the prioritised interventions was circulated to participants and submitted to the AMA Federal Council for formal endorsement.

The prioritisation process was evaluated for transparency, inclusivity, rigour and feasibility. Transparency and rigour were supported through structured facilitation, workshop observations and consensus achievement across Delphi rounds, whereas inclusivity was assessed through participant engagement and completion rates.

### 2.5 Reflexivity

The authors brought diverse professional perspectives to this study, including senior clinicians and academics (HT, JP), with HT bringing expertise in priority‐setting workshops; a senior researcher with expertise in qualitative methods (BG); and an early career researcher (TVN) with experience in implementation and qualitative research. Two authors (SC and SW) represented the AMA, contributing policy and medical leadership expertise; both are members of the overarching AWHL Steering Committee.

Consistent with a co‐production approach [35], the AMA partnered with the AWHL team from the outset to shape study objectives, recruitment strategies and data collection tools. Interviews were conducted by a research assistant with qualitative expertise and no prior relationship with the participants. Analysis was led by BG and conducted by a research assistant, with regular team discussions supporting reflexivity, critical reflection on interpretations and peer debriefing. Final analytic decisions were made by the research team to ensure the findings remained data driven [36].

### 2.6 Ethics Statement and Reporting

This study was approved by the Monash University Human Research Ethics Committee (Project ID 25097) and is reported in accordance with the REporting guideline for PRIority SEtting of health research (REPRISE; Table S2) [27].

## 3 Results

### 3.1 Participants

#### 3.1.1 Mixed‐Methods Research (Aim 1)

Seventeen medical leaders were interviewed, representing all states and territories, a range of AMA councils, committees and boards and both early (*n* = 5) and senior (*n* = 12) leadership levels. The member survey (*n* = 542) included respondents from all states and territories, spanning career stages from doctors in training to those retired from practice. More than 75% had never served on any federal and/or state or territory council, committee, task force or working group, providing a strong grassroots perspective. Table 1 provides a summary of participant demographic characteristics.

**TABLE 1:** Participant demographics.

	Medical leader interviews (*n*)	Member survey (*n*, %)^a^	Priority‐setting workshop (*n*)
Total number of participants	17	542	22
Gender			
Woman	13	304 (56%)	14
Man	4	217 (40%)	8
Prefer not to say	—	21 (4%)	—
Non‐binary/gender diverse^b^	—	—	—
Membership type			
GP and non‐GP specialists	13	349 (64%)	13
Doctors in training	4	68 (13%)	6
Medical students	0	0	1
Administration	0	3 (1%)	2
Academic	0	11 (2%)	0
Retired from practice	0	53 (10%)	0
Other	0	58 (11%)	0
State/territory			
New South Wales	4	115 (21%)	3
Queensland	3	97 (18%)	3
Victoria	4	138 (25%)	6
Western Australia	1	114 (21%)	2
South Australia	2	37 (7%)	4
Tasmania	1	19 (4%)	1
Northern Territory	1	6 (1%)	2
Australian Capital Territory	1	16 (3%)	1

Abbreviation: GP, general practitioner.

^a^ Proportions may not add up to 100% due to rounding.

^b^ Where data were examined across gender, the specific findings of non‐binary/gender diverse respondents were not reported. This was to maintain confidentiality given the sample size of non‐binary/gender diverse respondents was less than 5.

#### 3.1.2 Priority‐Setting Workshop (Aim 2)

Of the 54 individuals invited to participate in the priority‐setting workshop, 26 were women, and 28 were men. Thirty‐two were unavailable, including 12 women and 20 men. Twenty‐two participants completed the initial ranking survey (Round 1) and attended the workshop, including 20 in‐person and two online. Attendees included Federal Council members (*n* = 11) and Equity, Inclusion and Diversity Committee members (*n* = 11; Table 1).

### 3.2 Evidence Synthesis and Desktop Research (Aim 1)

Published elsewhere, evidence synthesis identified organisational strategies to advance women in leadership [21, 23]. Key enablers of organisational change spanned six domains: (i) leadership commitment and accountability; (ii) organisational processes and policies; (iii) organisational culture, awareness and engagement; (iv) support tools; (v) mentoring and networks; and (vi) leadership development.

Analysis of AMA member data, policies, reports and events demonstrated organisational commitment to advancing equity and leadership for women in healthcare. Since 2017, system‐ and organisational‐level changes have included establishment of the Equity, Inclusion and Diversity Committee; introduction of gender targets for Federal Council positions, committees and board; and funding to support breastfeeding mothers' participation in representative activities.

### 3.3 Mixed‐Methods Research (Aim 1)

Findings from interviews and the member survey were broadly consistent and identified factors supporting gender equity in leadership and opportunities across six domains (Table S3). The opportunity areas are summarised in Table 2 and these informed the subsequent consensus‐building and prioritisation process.

**TABLE 2:** Australian Medical Association (AMA) opportunity areas based on triangulated findings from medical leader interviews and member survey.

Internal opportunities *Increase the visibility and representation of women in leadership positions*: Increase diversity at senior leadership levels of the organisation. Diversity across gender, cultural and ethnic background and career stage (i.e., doctors in training, new fellows, general practitioners and non‐general practitioner specialists) should include meaningful participation and avoid tokenism. *Develop processes and strategies to promote respect and call out inappropriate behaviour*: Address problematic and intimidating behaviours. Insufficient action has been perceived towards calling out behaviour against women such as harassment and bullying. Any formal process designed to report inappropriate behaviour should be promoted widely and role‐modelled by those in positions of power. Formal organisational processes—practised and supported—empower people to respond and report appropriately. *Develop and support policies that provide flexibility within the organisation's leadership positions*: Flexibility and family‐friendly practices have contributed to the changing nature of engagement within the organisation. Flexibility may include job sharing, part‐time positions, offline work, reduced time commitment for roles and meaningful engagement that may fluctuate. This would facilitate engagement of women by acknowledging competing pressures of balancing work, volunteer positions and family commitments. *Increase awareness of the organisation's commitment and strategies towards gender equity*: Although medical leaders referred to processes and policies that create organisational accountability for gender equity, many members were unaware of these efforts. *Training to facilitate mentors/mentees*: There was mixed support for the organisation to facilitate further mentoring and networking, given many external opportunities and variable success with formal programs. However, evidence suggests informal mentoring is vital. The organisation could offer training sessions to facilitate mentors/mentees rather than formal programs. There was strong support from members for mentoring and networking opportunities. *Advocate for and support the career development and engagement of women members*: This includes enabling equitable involvement of early career doctors in committees and councils, which provides an opportunity for developing leadership experience and professional networks. *Ensure leadership development programs are accessible*: Accessible leadership programs enable the participation of members who are parents and/or carers. Caring responsibilities increase inequity, with women predominantly affected. Access considerations include cost, timing and mode of delivery (e.g., synchronous training with others at a set time or asynchronous training at an individual's own pace). *Address the tension between merit and diversity to support implementation of gender targets and other gender equity strategies*: Methods to demonstrate that these approaches are not tokenistic and do enable qualified women to be appointed to positions should be explored.
Beyond the organisation to the broader medical profession *Advocate for the visibility of women in leadership across the medical profession*: Seeing more women in leadership across medicine is important. The organisation has an opportunity to advocate for and promote gender equity in leadership as a priority across the medical profession. This should include collaboration with medical colleges. *Address inequitable challenges faced by women across the medical profession*: This includes advocating for flexible training and working arrangements such as robust parental leave procedures and expression of availability for part‐time posts. *Advocate for and support the career development and engagement of medical women at all levels of training and work*: This includes supporting women at career junctures (e.g., career breaks, parental leave and returning to work). Advocating for pay equity is also vital.

### 3.4 Priority‐Setting (Aim 2)

#### 3.4.1 Round 1: Initial Delphi Survey Ranking, Pre‐Workshop

Results of the initial ranking are shown in Table 3. For *internal priorities*, the survey included 26 items to be ranked by importance, along with an ‘Other’ option for additional suggestions. Five participants each contributed one additional item. For *external priorities*, the survey included 16 items with a similar ‘Other’ option. No additional suggestions were submitted.

**TABLE 3:** Initial Delphi ranking survey findings.

**Top 10 ranked *internal interventions* for advancing gender equity in leadership** Of 26 initial items, these were the top 10 ranked: Involvement of early career women in Australian Medical Association (AMA) committees and councils. Monitoring, evaluating and reporting on implementation and progress toward gender equity and diversity within the AMA. Transparent and diverse decision‐making processes, including panels that have diversity across gender, cultural background and career stage. Promote and provide information on evidence‐based leadership development programs to AMA members. Training on gender bias, implicit bias and organisational systemic challenges that reach beyond the individual level. Actionable processes and strategies to promote respect and call out, report and monitor inappropriate behaviour in AMA meetings and events. Succession planning and sponsorship (support) for emerging women leaders to apply for AMA roles. Provide scholarships or funding to support leadership training and development for members on less income (e.g., junior and early career doctors, women, those working part‐time). Equitable recruitment and selection policies for leadership and representative roles where diversity and the principle of relative opportunity are considered across gender, cultural and ethnic background and career stage. Promote a clearer understanding of merit, including the influence of bias, privilege and preference for historical norms. (Equal 10th) Transparent and equitable processes for election to, acquiring and nomination for AMA leadership positions. Additional suggestions made by participants: Family‐friendly meeting times. Collaborative approach to scheduling committee meetings (e.g., survey committee members for best time, etc.). Promote mentoring for all genders (not just women mentoring women). Raise awareness of past harms to women and highlight affirmative action as catching up to correct past bias and discrimination. Alter the Constitution to that of AMA Victoria.
**Top 10 ranked *external interventions* for advancing gender equity in leadership** Support policy and work with medical colleges and health services to: Deliver on equal pay. Support policy and work with medical colleges and health services to: Implement strategies that enable access to parental leave for doctors across all genders. Support policy and work with medical colleges and health services to: Implement strategies and leverage tools that enable and support the career development and engagement of medical women across career junctures such as career breaks, parental leave and returning to work. Support policy and work with medical colleges and health services to: Implement strategies and leverage tools that enable flexible working arrangements, including availability for part‐time training posts. Advocate for and promote: Employers and colleges to provide opportunities for leadership programs across the medical profession. The AMA should take a strong, committed leadership position to promote a culture of gender equity in the medical profession. Support policy and work with medical colleges and health services to: Monitor and report on gender equity in leadership across medicine. Advocate for and promote: Gender equity in leadership as a priority across the medical profession. Advocate for and promote: Visibility of women in leadership across the medical profession. The AMA should take a strong, committed leadership position on gender equity in the medical profession.

#### 3.4.2 Round 2: Priority‐Setting Nominal Group Technique Workshop, Sense‐Making and Importance Delphi Survey Voting

Group discussion resulted in consolidation of intervention strategies considered similar or involving comparable strategic actions. Results of the Round 2 individual, confidential poll, where participants ranked interventions previously identified as ‘most important’, are summarised in Table 4.

**TABLE 4:** Summary of Round 2 voting on importance and Round 3 voting on feasibility after sense‐making and group discussion.

	Round 2 voting on importance	Round 3 voting on feasibility
Top ranked *internal interventions* for advancing gender equity in leadership	Embedding gender equity education on merit, bias and privilege and why gender equity is needed. Evidence‐based leadership, mentoring and support for emerging women leaders, including through scholarships/funding for leadership development. Transparent and equitable processes for decision‐making around recruitment, selection and acquiring leadership and representative roles, including by setting gender targets. Monitoring, evaluation and transparent reporting. Promote respect and monitor behaviour in AMA meeting/events.	Embedding gender equity education on merit, bias and privilege and why gender equity is needed. Transparent and equitable processes for decision‐making around recruitment, selection and acquiring leadership and representative roles, including by setting gender targets. Monitoring, evaluation and transparent reporting. Promote respect and monitor behaviour in AMA meeting/events. Evidence‐based leadership, mentoring and support for emerging women leaders, including through scholarships/funding for leadership development.
Top‐ranked *external interventions* for advancing gender equity in leadership	Career junctures, transitions and flexibility: Implement and leverage strategies to support career development including career junctures, parental leave for all, flexible work and part‐time training. The AMA should take a strong, committed leadership position promoting a culture of gender equity in the medical profession. Advocate for gender equity in leadership as a priority across the medical profession using a co‐design and stakeholders' approach, including considering the feminisation of medical sectors, working with policymakers and gender targets. Recognition for training and participating as a mentor (e.g., continuing professional development points). Strategies to deliver on the gender pay gap. Advocate for the robust monitoring and reporting of diverse metrics which will advance gender equity in leadership across the medical profession, including leadership at all levels.	The AMA should take a strong committed leadership position promoting a culture of gender equity in the medical profession. Advocate for gender equity in leadership as a priority across the medical profession using a co‐design and stakeholders' approach, including considering the feminisation of medical sectors, working with policymakers and gender targets. Advocate for the robust monitoring and reporting of diverse metrics which will advance gender equity in leadership across the medical profession, including leadership at all levels. Career junctures, transitions and flexibility: Implement and leverage strategies to support career development including career junctures, parental leave for all, flexible work and part‐time training. Recognition for training and participating as a mentor (e.g., continuing professional development points). Strategies to deliver on the gender pay gap.

Abbreviation: AMA, Australian Medical Association.

#### 3.4.3 Round 3: Final Feasibility Voting

In the final poll, participants ranked interventions previously identified as ‘most important’ based on feasibility. Feasibility voting shifted several priorities, particularly among external interventions; strategies related to AMA leadership, advocacy and monitoring rose in ranking, whereas career flexibility and transition‐related strategies ranked lower (Table 4).

Although six external priorities remained highly ranked following Rounds 2 and 3, the final consensus discussion consolidated these into five external priorities for inclusion in the consensus recommendations.

Workshop outcomes identified 10 final consensus‐based priorities (Table 5; Table S4). Five focused on implementation *within* the AMA and five on support and advocacy across the broader medical profession.

**TABLE 5:** Final list of prioritised, consensus‐based interventions for implementation internally (organisation‐level) and externally (system‐level) mapped to evidence‐informed domains.

Final priorities	Relevant evidence‐informed domains^a^
*Internal*—within the AMA (organisation‐level)	
*Embed equity into activities and education*: Generalised embedding into all AMA activities and targeted education on merit, bias and privilege and why gender equity is needed.	1, 3
2 *Fair and transparent leadership selection*: Transparent and equitable processes for decision‐making around recruitment, selection and acquiring leadership and representative roles, including by setting gender targets.	1, 2, 4
3 *Accountability and transparent reporting*: Monitoring, evaluating and reporting transparently.	1, 3, 4
4 *Respectful engagement and behaviour*: Promote respect and monitor behaviour at AMA meetings/events.	2, 3
5 *Invest in emerging women leaders*: Evidence‐based leadership, mentoring and support for emerging women leaders, including through scholarships/funding for leadership development.	4, 5, 6
*External*—support and advocacy for the broader medical profession (system‐level)	
*Champion gender equity in medicine*: Strong, committed leadership position promoting a culture of gender equity in the medical profession.	1, 3
2 *Advocate for monitoring and reporting of data*: Advocate for the robust monitoring and reporting of diverse metrics which will advance gender equity in leadership across the medical profession, including leadership at all levels.	1, 3, 4
3 *Build inclusive career pathways*: Career junctures, transitions and flexibility. Implement and leverage strategies to support career development, including career junctures, parental leave for all, flexible work and part‐time training.	2
4 *Reward mentorship via professional recognition*: Recognition for training and participating as a mentor (e.g., through continuing professional development points and similar frameworks).	5, 6
5 *Advocate for pay equity*: Strategies to deliver on the gender pay gap.	1, 2

Abbreviation: AMA, Australian Medical Association.

^a^ Domains key: 1 = leadership commitment and accountability; 2 = organisational processes, practices and policies; 3 = organisational culture, awareness and engagement; 4 = organisational support tools; 5 = mentoring and professional networks; 6 = leadership training and development.

## 4 Discussion

This study generated evidence‐informed, contextually relevant priorities for advancing gender equity in medical leadership through collaboration among academics, doctors, members and organisational leaders. A structured priority‐setting process enabled medical leaders to identify feasible interventions aligned with organisational strategy and member priorities.

Internal priorities focused on embedding gender equity into organisational structures and culture, including fair and transparent leadership selection, promoting and monitoring respectful behaviours and investing in emerging women leaders. External priorities leveraged the organisation's national influence to advocate for systemic reform, including championing gender equity in medical leadership, data‐driven advocacy, inclusive career pathways, rewarding mentoring and pay equity.

Consistent with the opportunity areas (Table 2), participants highlighted barriers related to the culture of medicine and the organisation, including concerns regarding merit and diversity initiatives, limited visibility of women leaders, low awareness of existing gender equity efforts and experiences of inappropriate behaviour, such as intimidation, bullying and harassment. These findings suggest that advancing gender equity requires attention to both organisational structures and culture. Member responses revealed differing views regarding gender equity strategies, particularly tensions between merit‐based selection and gender targets. Although some viewed targeted approaches as necessary to address systemic barriers to women's leadership, others expressed concerns about reverse discrimination and perceived departures from merit‐based processes. Addressing these concerns, increasing awareness of existing efforts and communicating how targeted strategies are intended to complement merit‐based selection may support implementation. These findings highlight the structural and cultural challenges of advancing gender equity and the need for ongoing engagement, education and dialogue.

Despite achieving gender parity in medical schools more than a decade ago, women remain under‐represented in several medical and surgical specialties [37] and in senior leadership roles [1, 2, 18]. This under‐representation reflects systemic barriers rather than individual choice or capability, with organisational culture and practices shaping women's leadership opportunities and experiences. System‐ and organisational‐level interventions are important to address entrenched barriers and support inclusive leadership pathways [19, 21].

Accelerating gender equality in healthcare leadership requires a shift from individual responsibility to collective action [4, 19] through coordinated, evidence‐informed strategies involving policymakers, healthcare organisations, academic institutions and professional bodies [35]. Collaborative reform can help create environments that enable and sustain women's leadership across the healthcare system [38].

This study demonstrates how implementation science methods can identify priorities for change and, to our knowledge, is among the first to examine medical leadership at both system and organisational levels.

This study produced consensus‐based priorities to inform a co‐produced implementation plan for gender equity in leadership within the AMA, including an action plan and evaluation strategy to advance gender equality within the AMA and across the medical profession. The research team is supporting implementation through capacity‐building activities designed to strengthen the AMA's capability to implement, monitor and sustain change.

Professional associations and specialist colleges may be particularly well‐positioned to influence systemic change through advocacy, policy development, standard‐setting and negotiation of industrial agreements. Embedding gender equity into organisational governance and leveraging national influence can support progress toward inclusive leadership. Importantly, the culture and expectations set by a professional member organisation can shape both organisational and member practice within clinical and professional contexts. These priorities offer a roadmap for moving beyond ad hoc initiatives toward structured, evidence‐informed action.

Although consensus‐based priority setting provides a foundation for change, achieving gender equality in leadership will require sustained leadership commitment, resources, organisational capacity, member engagement and ongoing evaluation. Implementation may also be influenced by competing priorities and changing external contexts. This study represents the first phase of a broader implementation and evaluation program. The Centre is supporting implementation and evaluating impacts on leadership representation, organisational culture and workforce outcomes. Comparative research across health sector organisations and jurisdictions is underway to identify best practices and inform national strategies. Longitudinal evaluation will be critical to assess sustainability and long‐term effects.

### 4.1 Strengths and Limitations

A key strength of this study is its application of implementation science to priority‐setting. Integrating implementation frameworks, evidence synthesis, contextual assessment and co‐production with organisational leaders and members generated feasible, actionable priorities. The structured consensus process enhanced transparency, inclusivity and contextual relevance, while facilitation by experienced researchers maintained neutrality and supported interest‐holder‐led decision‐making.

Participants were purposively selected organisational leaders and included a higher proportion of women than the broader membership. Consequently, findings may reflect perspectives of individuals more engaged in gender equity initiatives. Because voting was anonymous and gender was not collected at the individual level, gender‐stratified voting patterns and consensus outcomes could not be examined. Findings are derived from a single professional association and reflect its specific context and member priorities. Although many participants also belong to specialist colleges, results pertain to their roles and engagement within the AMA.

Although findings may not be directly transferable to other contexts, the methods provide a scalable framework for application across health services and organisations. The AWHL team has applied the same process with additional professional colleges and health services; findings from these settings will be reported separately.

## 5 Conclusion

This study demonstrates that structured implementation science methods can co‐produce evidence‐informed priorities for advancing gender equity in medical leadership. Through engagement with academics, doctors, members and organisational leaders, the process identified strategies aligned with organisational goals and was designed to strengthen commitment to change. The findings offer a scalable framework for professional associations and health organisations seeking collaborative, sustainable, system‐level change.

## Author Contributions

H.J.T., B.G., S.C., and S.W. contributed to the conceptualisation of the study. Methodology was developed by B.G., J.P. and H.J.T. Formal analysis was undertaken by B.G., J.P. and T.V.N. Investigation was carried out by B.G., J.P., T.V.N., S.C., S.W. and H.J.T. Data curation was managed by T.V.N. The original draft was prepared by B.G., with authors J.P., S.C., S.W. and H.J.T. contributing to review and editing. Project administration was led by B.G., while supervision and funding acquisition were provided by H.J.T.

## Funding

This research was supported by funding from two National Health and Medical Research Council (NHMRC) Partnership Grants (APP1191837 and 2018718) and partner contributions. The NHMRC had no role in the design, conduct, analysis, interpretation or reporting of this study. The Australian Medical Association did not provide direct financial funding for this study but did contribute to the overall partnership project. Helena J. Teede receives funding from an NHMRC Fellowship.

## Disclosure

Not commissioned; externally peer‐reviewed.

## Conflicts of Interest

The authors declare no conflicts of interest.

## Supporting information


**Table S1:** AMA priority setting workshop: Feasibility Framework.
**Table S2:** REPRISE Compliance Checklist.
**Table S3:** Summary of triangulated medical leader interviews and member survey findings.
**Table S4:** Final list of consensus‐based priorities for implementation.

## Data Availability

Data analysed in this study are not publicly available to protect participant confidentiality and in accordance with the conditions of the approved ethics protocol.
