# Supplementary material for: Driving Implementation‐Focused Change for Gender Equity in Medical Leadership: A Consensus‐Based Priority‐Setting Approach
Source: Med J Aust. 2026 Oct 6;224(10):e70314. doi: 10.5694/mja2.70314 (PMC13642974; doi:10.5694/mja2.70314)
Supplement: Supplementary file 1 [file MJA2-224-0-s001.pdf]

MJA
The Medical Journal of Australia

Supporting Information

Supplementary material

This appendix was part of the submitted manuscript and has been peer reviewed.
It is posted as supplied by the authors.

Appendix to: B. Garth, J. Proimos, T. V. Nguyen, et al. Driving Implementation-Focused Change for Gender Equity in Medical Leadership: A Consensus-Based Priority-Setting Approach. *Medical Journal of Australia* 2026; doi: 10.5694/mja2.70314.

SURVEY S1. Modified Delphi survey (Round 1).

Introduction

INTRODUCTION

The AMA is partnering with the Advancing Women in Healthcare Leadership (AWHL) project and is seeking your views through this online survey to inform the AMA AWHL priority setting workshop on 3 August 2023. The survey is the first of two parts in a formal process that aims to inform priorities for implementing evidence-based gender equity interventions at the AMA. The top interventions identified from this survey will be taken to the priority setting workshop in August.

Gender equity interventions included in this survey are extracted from three sources:

1. A recent systematic review of cross sector evidence on organisational activities that **advance women in leadership.**
2. Interview data from **AMA leaders** with diversity across gender, location, and AMA councils, committees and boards.
3. Survey data from **AMA members** with diversity across gender, age, location and membership type.

In this survey, we are interested in what you **believe to be important** and **acceptable** from a full suite of evidence-based interventions.

- The survey should take no longer than 15 minutes
- Responses are anonymous

- You can withdraw at any time. After you have submitted your survey it cannot be retrospectively removed because responses are anonymous.
- Please read this Participant Information Sheet before deciding to take part

By clicking the button below, you acknowledge:

- You have read the Participant Information Sheet provided
- You understand the purpose, procedures and risks of the research project
- You have had the opportunity to ask questions (mchri.awhl@monash.edu) and are satisfied with the answers you received
- You freely agree to participate in this research project as described in the Participant Information Sheet

○ I consent, begin the survey

○ I do not consent, I do not wish to participate

Context

CONTEXT

Before we start, let's quickly review the work undertaken that brings us to this point.

FIRSTLY | Evidence from the literature

Twenty years of research was comprehensively, systematically reviewed to capture evidence on effective organisational strategies that effectively advance women in leadership.[1,2] *Organisational leadership, commitment and accountability* were necessary for

organisational change, in addition to key strategies organised under 5 main categories (Figure 1).

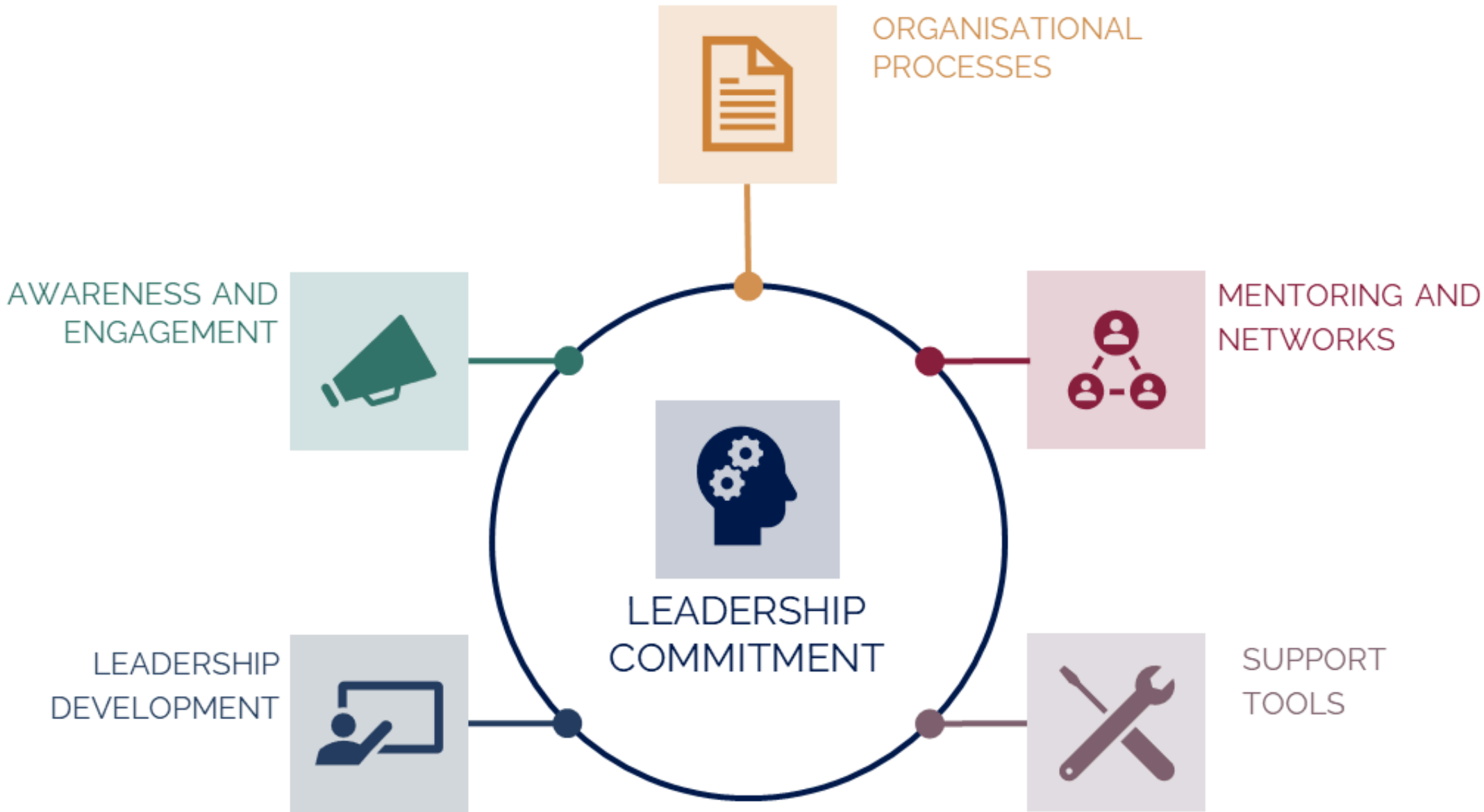

Figure 1. Effective organisational strategies for advancing women in leadership

SECONDLY | Evidence from the AMA

Informed by key findings from the literature, the Advancing Women in Healthcare Leadership (AWHL) team from Monash University partnered with the AMA to identify effective organisational strategies for advancing women in leadership roles at the AMA and across the medical profession. The aim here was to inform AMA's future direction for achieving gender equity in healthcare leadership. **Interviews with 17 AMA leaders** and a **survey of AMA members (completed by 542 members)** identified what works to advance women in healthcare leadership – internally to the AMA and externally to the medical profession – and key opportunities for consideration. The results are included in the AMA AWHL Progress Report May 2023.

NOW | Opportunities for the AMA

Based on the rigorous, evidence-based research described, numerous interventions to advance women in healthcare leadership were identified. **In this survey** you have the important task of nominating which interventions **you think the AMA should prioritise** for delivering gender equity outcomes 1/ at the AMA and 2/ beyond the AMA to the medical profession.

AFTER | Next steps

- Findings will be compiled and presented at the workshop on 3 August
- At the workshop, attendees will go through a process of discussing the top nominated interventions, and ranking interventions according to their importance and feasibility.
- The outcome will be AMA's agreed priorities for 1/ within the AMA and 2/ beyond the AMA, across the health sector.

SECTION 1: INTERNAL AMA

SECTION 1: INTERNAL AMA

Select ALL interventions you would prioritise as most important for delivering gender equity outcomes that advance women into leadership *<u>WITHIN THE AMA</u>*

Organisational processes

- [ ] Transparent and equitable processes for election to, acquiring and nominating for AMA leadership positions

- ☐ Transparent and diverse decision-making processes, including panels that have diversity across gender, cultural background and career stage
- ☐ Succession planning and sponsorship (support) for emerging women leaders to apply for AMA roles
- ☐ Involvement of early career women in AMA committees and councils
- ☐ Flexibility in selection requirements for leadership and representative roles, e.g., removal of required long-lead time and multiple volunteer positions for leadership positions
- ☐ Flexibility in time commitment for leadership and representative roles
- ☐ Limited tenure in leadership roles to avoid perpetuation of exclusivity in leadership
- ☐ Gender equity targets, i.e., aspirational goals
- ☐ Gender equity quotas, i.e., mandatory outcomes

Awareness and Engagement

- ☐ Actionable processes and strategies to promote respect and call out, report and monitor inappropriate behaviour in AMA meetings and events
- ☐ Promote a clearer understanding of ‘merit’, including the influence of bias, privilege and preference for historical norms
- ☐ Raise awareness that merit-based assessments are subject to bias and different opportunity and privilege, and ensure formal awards, celebrations, and acknowledgements of member achievements are free from gender bias, are transparent, and are assessed relevant to opportunity
- ☐ Training on gender bias, implicit bias, and organisational systemic challenges that reach beyond the individual-level
- ☐ Explicit acknowledgement of past harms and discrimination faced by women in medicine
- ☐ Explicit internal and external communication of the AMA’s commitment towards gender equity in leadership
- ☐ Explicit internal and external communication promoting AMA’s commitment to respectful inclusive behaviours

Support tools

- ☐ Transparent position descriptions for AMA leadership and representative roles, i.e., define time and additional commitment requirements
- ☐ Equitable recruitment and selection policies for leadership and representative roles where diversity and the principle of relative to opportunity is considered across gender, cultural and ethnic background, and career stage
- ☐ Monitoring, evaluating and reporting on implementation and of progress toward gender equity and diversity within the AMA

Mentoring and Networking

- ☐ Training sessions to facilitate mentoring and networks
- ☐ Promotion of mentoring for women
- ☐ An embedded mentorship program for women that aims to facilitate and support women to obtain mentors
- ☐ Facilitate professional networking events for women

Leadership development

- ☐ Provide scholarships or funding to support leadership training and development for members on less income, e.g., junior and early career doctors, women, those working part-time
- ☐ Promote and provide information on evidence-based leadership development programs to AMA members
- ☐ Explicit internal and external communication promoting AMA's commitment to respectful inclusive behaviours

Other

- ☐ [ ] Others (please specify)

SECTION 2:

SECTION 2: IMPLEMENTATION OF GENDER EQUITY INTERVENTIONS WITH AMA

The following questions are focused on the implementation of prioritised gender equity interventions with AMA

	Not at all capable	Not so capable	Somewhat capable	Very capable	Extremely capable
How would you rate the AMA's current **capability** to implement gender equity interventions?	○	○	○	○	○

Explain:

Click to write the question text

	Not at all ready	Not so ready	Somewhat ready	Very ready	Extremely ready
How would you rate the AMA's **readiness** to implement gender equity interventions?	○	○	○	○	○

Explain

Click to write the question text

What level of **support** do AMA members need from the AMA and its leaders to adopt and embrace gender equity interventions?	None at all	A little	A moderate amount	A lot	A great deal
	○	○	○	○	○

Explain

Click to write the question text

What level of **knowledge** do you believe the **AMA** has in relation to **implementing** gender equity interventions?	None at all	A little	A moderate amount	A lot	A great deal
	○	○	○	○	○

Explain

Block 7

SECTION 3: EXTERNAL AMA

The following opportunities align with the AMA's broader commitment to promoting equity, inclusion and diversity in the AMA and the medical profession more broadly. In its role as the peak professional body for doctors in Australia – representing doctors and working with governments to develop and influence health policy to provide the best outcomes for doctors, their patients and the community – the AMA will set priorities that relate to its remit across the medical profession.

Select ALL interventions you believe are most important for the AMA, to contribute to delivering gender equity outcomes that advance women into leadership *EXTERNAL TO THE AMA* and across the medical profession

Leadership position:

- ☐ The AMA should take a strong committed leadership position to gender equity in the medical profession (awareness and engagement)
- ☐ The AMA should take a strong committed leadership position to advocate for the involvement of women in the medical workforce in engagement and co-design processes (awareness and engagement)

- ☐ The AMA should take a strong committed leadership position to promote a culture of gender equity in the medical professional (organisational processes)
- ☐ The AMA should take a strong committed leadership position in engagement of policy makers and broader stakeholders on gender equity (awareness and engagement)

Advocate for and promote:

- ☐ Gender targets (i.e. aspirational goals) across the medical profession (organisational processes)
- ☐ Gender quotas (i.e. mandatory outcomes) across the medical profession (organisational processes)
- ☐ Visibility of women in leadership across the medical profession (awareness and engagement)
- ☐ Gender equity in leadership as a priority across the medical profession (awareness and engagement)
- ☐ Mentoring across the medical profession (mentoring and networking)
- ☐ Employers and colleges to provide opportunities for leadership programs across the medical profession (support tools)

Support policy and work with medical colleges and health services to:

- ☐ Monitor and report on gender equity in leadership across medicine (support tools)
- ☐ Support the career development and engagement of medical women at all levels of training and work (awareness and engagement; leadership development)
- ☐ Implement strategies and leverage tools that enable and support the career development and engagement of medical women across career junctures such as career breaks, parental leave and returning to work (organisational processes; support tools)
- ☐ Implement strategies and leverage tools that enable flexible working arrangements including availability for part-time training posts (organisational processes; support tools)
- ☐ Implement strategies that enable access to parental leave for doctors across all genders (organisational processes; awareness and engagement)

- ☐ Deliver on equal pay (support tools)

Other

- ☐ [ ] Others (please specify)

Section 4

SECTION 4: FOR INTERNAL AND EXTERNAL GENDER EQUITY ACTIVITIES

The following questions are focused on the enablers for the AMA to implement gender equity interventions

Select ALL items you believe are important for implementing gender equity for women at the AMA

- ☐ Case Studies - examples of WHAT has been successfully implemented previously
- ☐ Case Studies - examples of HOW to successfully implement gender equity interventions
- ☐ Promotion of evidence-based strategies on WHAT and HOW
- ☐ Specific examples of what works within the healthcare and other sectors
- ☐ Support for leadership and management in organisations to understand how to support this work
- ☐ Demonstration of how to include a gender equity intervention into a program, policy or service
- ☐ Demonstration of how to include a gender equity intervention into a major/large strategy (whole department, region, state)

- ☐ Guidance and tips on how gender equity interventions can be implemented across the organisation and become "business as usual"
- ☐ Tools to support implementation

Select ALL delivery options you believe are important for the delivery of these enablers

- ☐ Webinars sharing the process of how to implement gender equity interventions
- ☐ Small group peer support to share safely how the work is progressing
- ☐ Training - short sharp (1 to 2 hour) sessions on specific steps in the process
- ☐ Training - 1 to 2 days to go into detail about the process
- ☐ Conference sessions/workshops on gender equity and leadership training
- ☐ Improved access to gender equity resources via the AMA website

Are there any further resources, training or support that would assist you and the AMA?

Are there additional barriers or challenges that you or the AMA might experience when implementing gender equity interventions?

Section 5: Demographics

DEMOGRAPHICS

***We're almost there!* Please complete the last few questions.**

What is your membership type?

- ○ Doctor in training
- ○ GP and non-GP specialist
- ○ Retired from practice
- ○ Medical student
- ○ Academic
- ○ Administration
- ○ Other (please specify)

In what capacity are you contributing to this survey:

- ☐ AMA Board member
- ☐ AMA Federal Council member
- ☐ AMA Presidents and CEOs member
- ☐ AMA Equity, Inclusion and Diversity Committee member

☐ [ ] Other (please list)

What is your gender?

- ○ Woman
- ○ Man
- ○ Non-binary / gender diverse
- ○ My gender identity is not listed. I identify as: (please specify) [ ]
- ○ I prefer not to say

What is your age?

[▾]

In what State/Territory do you reside?

[▾]

Do you identify as an Australian Aboriginal and/or Torres Strait Islander person?

How would you describe your cultural background? (Please select up to 2 cultural backgrounds)

- ☐ Indigenous Australian
- ☐ Australian (excl. Indigenous Australian)
- ☐ New Zealander (not Maori)
- ☐ Maori, Melanesian, Papuan, Micronesian, and Polynesian
- ☐ Anglo-European
- ☐ North-West European (excl. Anglo-European)
- ☐ South-East European
- ☐ South-East Asian
- ☐ North-East Asian
- ☐ Southern and Central Asian
- ☐ North American
- ☐ South and Central American and Caribbean Islander
- ☐ North African and Middle Eastern
- ☐ Sub-Saharan African
- ☐ ____________ Other cultural/ethnic group, please specify
- ☐ Unsure
- ☐ Prefer not to say

Powered by Qualtrics

TABLE S1: AMA priority setting workshop: Feasibility Framework.

**Criteria 1 | Rationale |** *Evidence-base to improve outcomes*
- Is there a strong rationale/evidence-base for the proposed initiatives/interventions to improve outcomes for women?<br>- Is there evidence for the proposed approach to address a gap in current organisational practice at the AMA and beyond?<br>- Is it within the AMA's remit?
**Criteria 2 | Prevention |** *Preventing poor outcomes*
- Is there potential to prevent significant problems or secondary impacts for women at the AMA and across the medical profession? E.g. Can these priorities prevent poor career outcomes for women?<br>- Are these interventions better than other options or current practice?
**Criteria 3 | Location |** *Improving outcomes nationally*
- Consider geographical/regional issues for State/Territory AMA and Federal AMA. What are the inequities that can be improved by these interventions?<br>- Is there a need to adapt for States/Territories? E.g. Would these interventions improve outcomes for women in the medical profession across States/Territories, including across metropolitan, regional and rural areas? Would they impact vulnerable groups of women?
**Criteria 4 | Participation |** *Engagement needed to drive impact*
- Is there potential for strong engagement in the proposed interventions? E.g. is there a role/fit for these interventions across State/Territory and Federal AMA?<br>- Are there existing relationships between stakeholders that can be leveraged to support these interventions? Is the AMA networked with external entities?<br>- How complicated are the interventions in scope/nature?
**Criteria 5 | Policy |** *Alignment with policy*
- Does the problem or potential solution align with current policy directions at the AMA. E.g. Do gender equity initiatives align with policy priorities?
**Criteria 6 | Strategy |** *Alignment with strategic direction*
- Do the interventions align with the AMA's mission/strategic direction/ strategic priority areas?
**Criteria 7 | Transformation |** *Success and transformational change*
- Will addressing this problem/ taking this approach collaboratively, lead to a transformational change for women across the AMA and more broadly across the medical profession?<br>- Would this initiative transform the workforce and impact on care and outcomes?
**Criteria 8 | Financial considerations |** *Resourcing and benefits of implementing priorities*
- Are the interventions feasible to institute? Are they affordable?<br>- Are the costs and benefits of implementing the interventions considered worthy, compared to a situation where these interventions did not take place?<br>- Are there resources available to help implement these interventions?

TABLE S2. REPRISE Compliance Checklist.

While REPRISE was developed for health research priority-setting, we applied it because our study used a structured, stakeholder-driven, consensus-based prioritisation process in an organisational setting, consistent with REPRISE's flexible scope and endorsed methods including Delphi surveys and nominal group technique.

No	Item	Where reported in manuscript
**A**	**Context and scope**	
1	Define geographical scope	Abstract (Setting); Methods (Setting)
2	Define health area, field, focus	Abstract (Objectives); Introduction
3	Define the intended beneficiaries	Introduction
4	Define the target audience of the priorities	Introduction
5	Identify the research area	Abstract (Study type); Methods (Study design)
6	Identify the type of research questions	Introduction; Methods (Study design)
7	Define the time frame	Methods (Qualitative research & Priority-setting process)
**B**	**Governance and team**	
8	Describe the selection and structure of the leadership and management team	Methods (Setting, Study design, Reflexivity). Cited study protocol in Study design.
9	Describe the characteristics of the team	Methods (Reflexivity)
10	Describe any training or experience relevant to conducting priority setting	Methods (Reflexivity)
**C**	**Framework for priority setting**	
11	State the framework used (if any)	Methods (Study design)
**D**	**Stakeholders or participants**	
12	Define the inclusion criteria for stakeholders involved in priority-setting	Methods (Participant recruitment)
13	State the strategy or method for identifying and engaging stakeholders	Methods (Participant recruitment, Data collection)
14	Indicate the number of participants and/or organisations involved	Results (Participants)
15	Describe the characteristics of stakeholders	Results (Participants)
16	State if reimbursement for participation was provided	Methods (Participant recruitment)
**E**	**Identification and collection of research priorities**	
17	Describe methods for collecting initial priorities	Methods (Modified Delphi survey, round 1)
18	Describe methods for collating and categorizing priorities	Methods (Modified Delphi survey, round 1)
19	Describe methods and reasons for modifying (removing, adding, reframing) priorities	Results (Priority-setting workshop, round 2)
20	Describe methods for refining or translating priorities into research topics or questions	Results (Priority-setting workshop, rounds 1 & 2)
21	Describe methods for checking whether research questions or topics have been answered	Methods (Study design)

22	Describe number of research questions or topics	Results (Round 1, Initial ranking, pre-workshop)
**F**	**Prioritisation of research topics/questions**	
23	Describe methods and criteria for prioritizing research topics or questions	Methods (Modified Delphi survey and NGT workshop)
24	State the method or threshold for excluding research topics/ questions	Methods (Priority-setting process, round 1, 2, 3), Results (Priority-setting workshop, round 1)
**G**	**Output**	
25	State the approach to formulating the research priorities	Results (Priority-setting workshop, round 3)
**H**	**Evaluation and feedback**	
26	Describe how the process of prioritisation was evaluated	Methods (Priority-setting process)
27	Describe how priorities were fed back to stakeholders and/or to the public; and how feedback (if received) was addressed and integrated	Methods (Priority-setting process)
**I**	**Implementation**	
28	Outline the strategy or action plans for implementing priorities	Discussion & Conclusion
29	Describe plans, strategies, or suggestions to evaluate impact	Discussion
**J**	**Funding and conflict of interest**	
30	State sources of funding	Title page
31	Declare any conflicts or competing interests	Title page

TABLE S3. Summary of triangulated medical leader interviews and member survey findings.

<table>
<tr><th></th><th>DOMAIN</th><th>FACTORS SUPPORTING GENDER EQUITY IN LEADERSHIP</th><th>OPPORTUNITY AREAS</th></tr>
<tr><td>1</td><td>Leadership Commitment & Accountability</td><td>• Visible representation of women in leadership roles.
• Gender targets (40:40:20) and monitoring mechanisms that drive accountability.
• Supportive leaders who actively encourage and sponsor women.
• Succession planning that identifies and develops emerging women leaders.</td><td>• Increase diversity across senior leadership positions.
• Increase visibility of women in senior leadership roles.
• Improve transparency of election and appointment processes.
• Promote inclusive, collaborative leadership styles.
• Reduce barriers associated with entrenched networks and long tenure.</td></tr>
<tr><td>2</td><td>Organisational Processes, Practices & Policies</td><td>• Equity, inclusion and diversity governance structures.
• Family-friendly policies and supports.
• Flexible participation models, including virtual engagement.
• Inclusive recruitment and appointment processes.
• Leadership and committee opportunities that build experience and exposure.</td><td>• Expand flexible leadership pathways and participation options.
• Review leadership requirements that disadvantage those with caring responsibilities.
• Improve transparency of leadership pathways and expectations.
• Strengthen systems for preventing and addressing inappropriate behaviour.
• Enhance implementation support for policies and succession planning.
• Address tensions between merit and diversity initiatives.</td></tr>
<tr><td>3</td><td>Organisational Culture, Awareness & Engagement</td><td>• Greater representation of women in leadership contributing to improved culture.
• Gender targets that increase awareness and engagement.
• Flexible and family-friendly approaches that support inclusion.
• Broader societal momentum towards gender equity.</td><td>• Increase visibility of women across communications and leadership representation.
• Address exclusionary or “boys’ club” cultures.
• Strengthen reporting and accountability for inappropriate behaviour.
• Acknowledge the ongoing impacts of gender discrimination in medicine.
• Improve member awareness of gender equity initiatives.
• Monitor and communicate progress towards equity goals.</td></tr>
<tr><td>4</td><td>Organisational Support Tools</td><td>• Professional development and skills-building opportunities.
• Leadership, media and project-based learning opportunities.
• Childcare and carer supports that facilitate participation.
• Recognition and celebration of achievements.
• Promotion of women leaders and role models.
• Organisational advocacy for gender equity across the profession.</td><td>• Ensure gender-equitable and transparent nomination, recruitment and recognition processes.
• Strengthen support for culturally diverse women and intersectional approaches.
• Enhance monitoring and evaluation of gender equity outcomes.
• Increase visibility of leaders from diverse backgrounds.</td></tr>
<tr><td>5</td><td>Mentoring & Professional Networks</td><td>• Strong mentoring and networking culture.
• Informal mentoring relationships that provide guidance and support.
• Formal mentoring opportunities for those with fewer connections.
• Networking through committee and leadership participation.
• Connections between women leaders that support professional growth and role modelling.</td><td>• Provide mentor and mentee training.
• Increase early-career participation in committees and councils.
• Strengthen networking pathways for emerging women leaders.</td></tr>
<tr><td>6</td><td>Leadership Training & Development</td><td>• Leadership programs that build capability and confidence.
• Valued opportunities such as Future Leaders programs, media training and committee involvement.
• Development activities that foster networks and leadership exposure.
• Financial support that improves access to training.</td><td>• Improve accessibility through flexible delivery, timing and cost.
• Expand leadership development in communication, mentoring and inclusive leadership.
• Improve equitable access to training opportunities.
• Increase leadership experience opportunities for early-career doctors.</td></tr>
</table>

TABLE S4. Final list of consensus-based priorities for implementation.

	Top 5 INTERNAL Priorities for Advancing Gender Equity in Leadership	Description/annotations
1	**Embed equity: Generalised embedding into all Australian Medical Association (AMA) activities and targeted education on 'merit', bias and privilege and why gender equity is needed***<br><br>*Including increasing the diversity, equity and inclusion literacy of leaders and members.	Gender bias and inequity continues to negatively impact the career progression and choice of medical specialty for women. In order to support implementation of gender equity interventions within the AMA, it is important to improve awareness and literacy among AMA members of the evidence-based need for gender equity, and the ways in which bias (conscious or unconscious), privilege and merit can impact the inequities faced and opportunities afforded to individuals.<br><br>The concept of merit assumes everyone has an equal opportunity to develop their talents and skills. However, the playing field is uneven and individuals may experience limited opportunities due to structural inequalities, e.g., around gender, race, ethnicity, sexuality and socioeconomic status. Systemic barriers can prevent some individuals from fully developing their potential. Reframing merit through the lens of equity and inclusion means striving to determine an individual's past performance and future potential through objective examination and interrogation of bias, with consideration to inequities faced and opportunities given to individuals.
2	**Ensure fair and transparent leadership selection: Transparent and equitable processes for decision-making around recruitment, selection and acquiring leadership and representative roles, including by setting gender targets***<br><br>* The AMA has a gender target of 40 per cent women, 40 per cent men, 20 per cent flexible for all federal AMA councils and committees, with a gender diversity target of women holding 50 per cent of federal AMA representative positions overall for attainment by 2024.	Processes for attaining leadership positions can perpetuate gender bias. It is suggested to review the processes to ensure they are gender equitable, for:<br>1. Election to leadership positions<br>2. Acquiring leadership positions<br>3. Nominating for leadership positions<br>These should include transparent and gender equitable panels, and being assessed for positions relevant to opportunity. Positions on councils, committees and boards that require a long-lead time with multiple volunteer positions can exclude people who have less capacity (often women). Gender targets are an effective intervention for improving equity, and to further support their implementation it is important to demonstrate that these approaches are not tokenistic and do enable qualified women to be appointed to positions.
3	**Maintain accountability: Monitoring, evaluating and reporting transparently**	Use of measurement and evaluation tools are important to develop a culture of continuous learning and growth. While the AMA continues its work in diversity, equity and inclusion, monitoring, evaluating and reporting progress to members is important. In addition to enabling the AMA to track its progress and continually inform improvements, this would also promote the work being done toward solutions and demonstrate the AMA's leadership commitment and accountability toward advancing women in healthcare leadership.
4	**Embed respectful engagement: Promote respect and monitor behaviour in AMA meeting/events**	The nature of AMA leadership culture can deter people, including many women, from putting themselves forward for leadership positions. Promoting respect and monitoring behaviour in AMA meetings/events is important for contributing to a positive and collaborative organisational culture. Processes and strategies that promote respect and call out inappropriate behaviour are needed to address problematic and intimidating behaviours. Insufficient action has been perceived towards calling out behaviour against women such as harassment and bullying. Any formal process designed to report inappropriate behaviour should be promoted widely and role modelled by those in positions of power. Formal organisational processes – practiced and supported – empower people to respond and report appropriately.
5	**Invest in emerging women leaders: Evidence-based leadership, mentoring and support for emerging women leaders, including through scholarships/funding for leadership development**	Leadership development opportunities and being mentored are valuable, evidence-based strategies for supporting women in their career development. Supporting emerging women leaders through scholarships/funding to participate in leadership development opportunities in particular, would improve equity for women who typically earn less and are more likely to work part-time. Enabling equitable involvement of emerging women leaders and early career doctors in committees and councils provides an opportunity for developing leadership experience and professional networks.
	**Top 5 EXTERNAL Priorities for Advancing Gender Equity in Leadership**	
1	**Champion gender equity in medicine: Strong committed leadership position promoting culture of gender equity in the medical profession**	Twenty years of evidence tells us that to advance women in leadership, organisational leadership, commitment and accountability is necessary for driving change. The AMA has an opportunity for considerable influence and advocacy to improve gender equity and advance women in leadership across the medical profession.
2	**Advocate for data: Advocate for the robust monitoring and reporting of diverse metrics which will advance gender equity in leadership across the medical profession including leadership at all levels**	In its leadership role promoting and protecting the professional interests of doctors, the AMA has the opportunity to advocate for the importance of robust monitoring and reporting of diverse metrics across the medical profession. This would enable meaningful measurement of improvements in the numbers and participation of women in healthcare leadership. Compounding forms of discrimination are known to impact career progression, pay inequality, and the experience of sexual harassment and discrimination at work. Metrics to consider include

		gender, culture and ethnicity, disability and sexuality. The Diversity Council Australia provides useful resources.
3	**Build inclusive career pathways: Career junctures, transitions and flexibility. Implement and leverage strategies to support career development including career junctures, parental leave for all, flexible work and part-time training.**	The current system is disjointed and requires alignment between medical Colleges and health services around flexible work/training policies, interrupted training time, family leave in training and parental leave policies. Through its Enterprise Agreements, the AMA has a key role here. Advocating for and implementing systems change requires a collective and collaborative effort between Colleges, health services, government and the AMA. Support is also needed for women at career junctures (e.g., career breaks, parental leave and returning to work).
4	**Reward mentorship through professional recognition: Recognition for training and participating as a mentor e.g. through CPD points and similar frameworks**	Mentoring is an evidence-based strategy for advancing women in leadership. Continuing professional development (CPD) points and similar frameworks would work to recognise its value and motivate participation for doctors to become mentors for women.
5	**Advocate for pay equity: Strategies to deliver on the gender pay gap**	Advocating and delivering on pay equity is vital. Data from the Workplace Gender Equality Agency (WGEA) reports that every occupation and manager level in Australia has a gender pay gap in favour of men, where ‘the more senior the job role, and pay, the bigger the gender pay gap’. Despite making up three quarters of the healthcare workforce, women earn much less than men working in the same sector.
